# Understanding the microstructure of a core–shell anode catalyst layer for polymer electrolyte water electrolysis

**DOI:** 10.1038/s41598-023-30960-x

**Published:** 2023-03-15

**Authors:** Salvatore De Angelis, Tobias Schuler, Mayank Sabharwal, Mirko Holler, Manuel Guizar-Sicairos, Elisabeth Müller, Felix N. Büchi

**Affiliations:** 1 Electrochemistry Laboratory, Paul Scherrer Institute, 5232 Villigen PSI, Switzerland; 2 Swiss Light Source, Paul Scherrer Institute, 5232 Villigen PSI, Switzerland; 3 Electron Microscopy Facility, Paul Scherrer Institute, 5232 Villigen PSI, Switzerland; 4 Present Address: Department of Energy Conversion and Storage, Technical University of Denmark, 2800 Kgs. Lyngby, Denmark

**Keywords:** Nanoscale materials, Imaging techniques

## Abstract

Reducing precious metal loading in the anodic catalyst layer (CL) is indispensable for lowering capital costs and enabling the widespread adoption of polymer electrolyte water electrolysis. This work presents the first three-dimensional reconstruction of a TiO_2_-supported IrO_2_ based core shell CL (3 mg_IrO2_/cm_2_), using high-resolution X-ray ptychographic tomography at cryogenic temperature of 90 K. The high data quality and phase sensitivity of the technique have allowed the reconstruction of all four phases namely pore space, IrO_2_, TiO_2_ support matrix and the ionomer network, the latter of which has proven to be a challenge in the past. Results show that the IrO_2_ forms thin nanoporous shells around the TiO_2_ particles and that the ionomer has a non-uniform thickness and partially covers the catalyst. The TiO_2_ particles do not form a percolating network while all other phases have high connectivity. The analysis of the CL ionic and electronic conductivity shows that for a dry CL, the ionic conductivity is orders of magnitudes lower than the electronic conductivity. Varying the electronic conductivity of the support phase by simulations, reveals that the conductivity of the support does not have a considerable impact on the overall CL electrical conductivity.

## Introduction

Polymer electrolyte water electrolysis (PEWE) is considered a key technology for producing green hydrogen using renewable energies. PEWE offers high current density, high hydrogen purity and excellent dynamic response to intermittent power fluctuations, making it ideal for meeting the intermittent demands of solar or wind power^1^. However, PEWE is characterized by high operational and capital expenditures which combined with the scarcity of platinum group metal based electrode materials (Ir, Pt), currently limit the widespread commercialization of the technology^2^.

A typical PEWE cell is composed of two catalyst layers (CLs) that serve as the electrodes, which are deposited onto a polymer electrolyte membrane (PEM). Liquid water is fed from a flow field at the anode side and distributed through a titanium porous transport layer (PTL)^3,4^ until it reaches the anodic CL where the oxygen evolution reaction (OER) takes place. The corrosive acidic environment provided by the ionomer membrane (pH ∼ 0) and the high cell operating potential (> 2 V), impose the use of scarce and expensive catalyst materials, often platinum group metals such Ir or Ru. This not only increases the cost of the electrolyzer (increasing capital expenditures) but, due to the limited annual production of Ir, also limits the installable power capacity to ~ 2 GW a^-1^^1^. It is therefore essential to optimize the anodic catalyst layer, to reduce catalyst loading while maintaining high cell current density and efficiency.

Despite the high costs and limited availability, IrO_2_-based catalysts are among the most commonly employed materials due to their relatively high activity and long-term stability for the oxygen evolution reaction (OER)^5^. The employment of a support promotes the dispersion of the IrO_2_ nanoparticles, increasing the electrochemically active surface area and thereby reducing IrO_2_ loading. Titania (TiO_2_) is the most commonly used support because of its high electrochemical stability and low cost^5^.

A typical anodic CL is usually coated onto the electrolyte membrane, using an ink composed of the catalyst powder mixed with a proton conducting polymer (ionomer). Upon drying, the mixture forms a complex three-dimensional microstructure. For the water-splitting reaction to occur, electrons, protons, and water/oxygen need to be transported efficiently in the respective phases. The IrO_2_ particles catalyze the OER reaction and are responsible for the CL electrical conductivity. The ionomer acts as a binder and provides ionic transport of the produced protons. Finally, the pores in the CL ensure the two-phase transport of water and oxygen to and from the reaction sites.

Therefore, the characterization of all four phases (pore, ionomer, IrO_2_, TiO_2_) and respective networks is essential for future, rational CL designs aiming at low loading and high efficiency. The challenge of anodic CL microstructure analysis lies in the difficulties of achieving high-quality 3D reconstructions, considering the small feature size (< 1 µm^6^). While three-dimensional reconstructions of PTLs can be easily found in literature for both ex situ^3,7^ and operando studies^8^, only one recent publication can be found where a PEWE CL reconstruction was obtained^6^. In^6^, the authors used focused ion beam-scanning electron microscopy (FIB-SEM) to reconstruct a CL employing an unsupported Ir-Ru catalyst. However, the ionomer structure could not be resolved and the transport properties of the electrode were deduced by artificially introducing the ionomer phase. Furthermore, the reconstruction of the ionomer phase requires the use of transmission electron microscopic tomography^9^ but this results in a reduction in the domain volume, analyzed for the higher resolution. In the last decade, X-ray ptychographic computed tomography (PXCT) was proven effective for reconstructing the microstructure of porous media for many energy devices, from batteries^10,11^ to solid oxide cells^12–14^. PXCT offers a unique combination of high spatial resolution and high sensitivity to differences in material density^15^ making it ideal for the analysis of the multi-phase microstructure of the PEWE CL.

In this work, PXCT is used for the first time to investigate the microstructure of a PEWE catalyst layer, based on TiO_2_/IrO_2_ catalyst material. Capitalizing on the high spatial resolution, the high data quality and near-perfect contrast among the phases, we were able to spatially resolve all the phases in the CL including the ionomer, which has been a challenge for most other reconstruction techniques employed for both PEWE and polymer electrolyte fuel cells. The CL microstructure obtained using PXCT is characterized and the effective transport properties in the different phases are also presented. Using the 3D reconstructed microstructure, the impact of support material conductivity is investigated.

## Materials and methods

### Sample preparation

The samples used for the PXCT procedure were extracted from representative catalyst coated membranes (CCMs), routinely used for electrochemical characterizations^16^. For the fabrication of the CCM, an IrO_2_ supported on TiO_2_ OER catalyst (MA-292, Umicore®, 75% w.t Ir) and a Pt on carbon hydrogen evolution reaction (HER) catalyst (TEC10E50E, Tanaka Kikinzoku Kogyo) were used. Both IrO_2_ and TiO_2_ are in the rutile form, as shown by Pătru et al.^17^.

The anode catalyst ink was prepared by stepwise addition of ultrapure water, 2-propanol, and Nafion solution (aliphatic alcohol/water, EW = 1100 g/mol, DuPont) to the IrO_2_-TiO_2_ powder. The concentration of the Nafion solution was 5 wt. %. The ink mixture was homogenized for 45 min. A Nafion® membrane N115 (Chemours, USA) was employed. The Nafion ionomer content relative to the total weight of the catalyst was set to 11 wt%, as optimized by Bernt et al.^18^ for IrO_2_ on TiO_2_ catalysts.

The spraying procedure was performed using an automated benchtop coating system (ExactaCoat, SONO TEC Corporation, USA). The anodic target loading of 3 mg_IrO2_ cm^-2^ was achieved by monitoring the online loading based on a reference sheet. Subsequently, the CCMs were dried for 12 h under ambient conditions. An in-depth description of the automated spray coating process is reported in^16^.

From the anodic CL, a pillar with a quasi-octagonal cross-section was extracted using the lift-out technique^19^, performed using the Ga-ion beam of a focused-ion-beam/scanning electron microscope (FIB-SEM, Zeiss Nvision 40). The sample was extracted using a micro-manipulator in the FIB-SEM and transferred onto the OMNY pin^20^ for mounting on the beamline rotation stage. More details on the lift-out procedure can be found in Section S1 of the supplementary materials.

### X-ray ptychographic nanotomography and data analysis

The PXCT measurement was performed at the X12SA (cSAXS) beamline at the Swiss Light Source, Paul Scherrer Institut, Switzerland. To avoid potential beam damage and reduce the mechanical degradation of the ionomer, the experiment was carried out using the OMNY microscope^21^, at cryogenic temperature (90 K) and under vacuum (2 × 10^–8^ mbar).

Ptychographic projections were measured at 7.2 keV photon energy, each with a scanning field of view of 19 × 11 μm^2^. The scanning points follow a Fermat spiral trajectory with average 1.5 μm step size. At each scanning position, a coherent diffraction pattern was recorded 7.2 m downstream of the object using an in-vacuum Eiger detector with 75 × 75 μm^2^ pixel size at 0.1 s exposure time. The illumination was defined by a gold Fresnel zone plate with 60 nm outermost zone width and a diameter of 220 μm, with the sample placed 2.4 mm downstream from the focal spot. Diffraction patterns with sizes of 1000 × 1000 pixels were used in the ptychographic reconstruction, giving an object pixel size of 16.4 nm. The ptychography reconstructions were carried out using the difference-map algorithm with a maximum likelihood refinement step^22^ using the PtychoShelves package^23^. For the tomogram, 1550 projections equally spaced over an angular range of 180° were collected. Three-dimensional ptychographic reconstruction were obtained through post-processing and fine alignment of the reconstructed 2D projections and a modified filtered back projection algorithm^24^.

**Figure 1:**
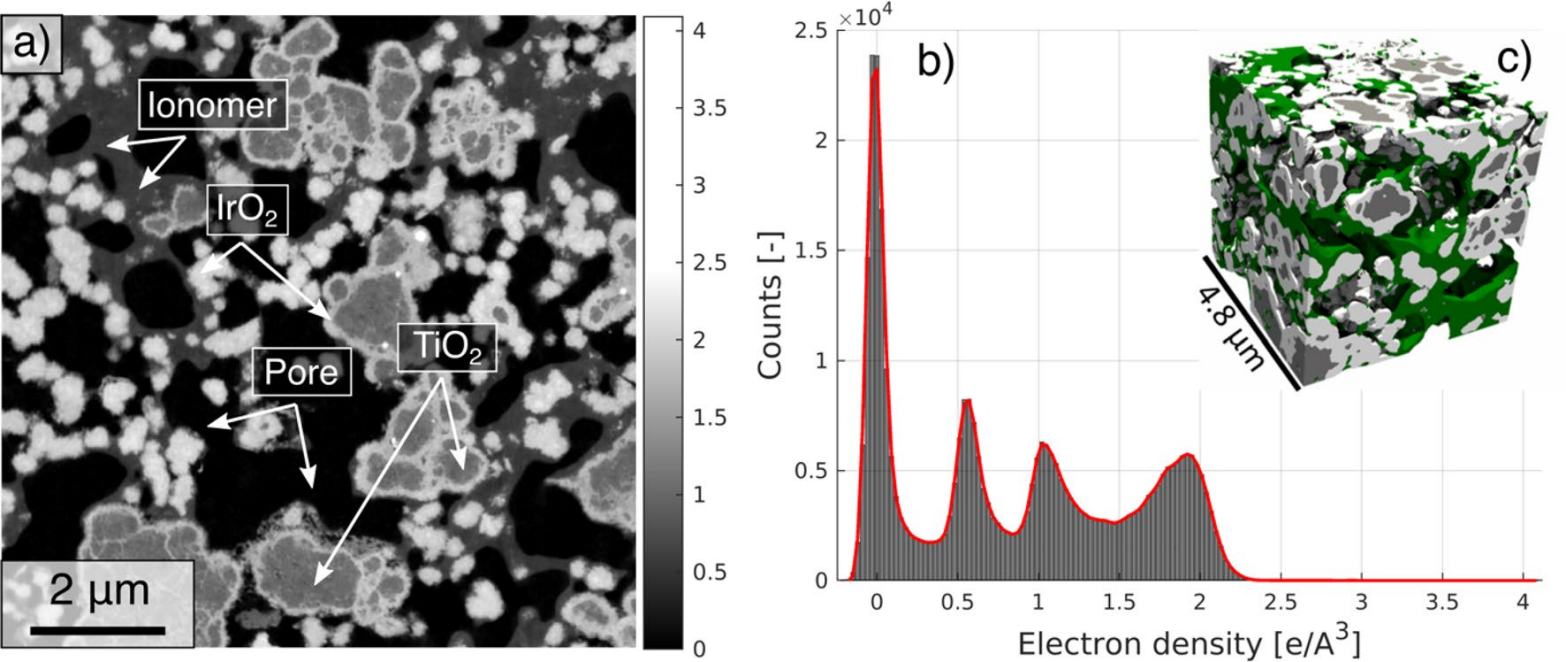
(**a**) Two-dimensional slice obtained from the entire tomogram showing all phases present in the electrode. In (**a**), the grayscale values represent the electron density expressed in e/A^3^. (**b**) Electron density histogram calculated over the entire volume; (**c**) Three-dimensional rendering of a subvolume extracted from the full segmented dataset. In (**c**), the white phase is the IrO_2_, the gray phase is the TiO_2_, the green phase is the ionomer and the porosity is transparent. The 3D rendering in (**c**) was made in Paraview 5.10 (Kitware, Inc., https://www.paraview.org).

The three-dimensional dataset was segmented using a 2D histogram thresholding procedure, which involves the determination of segmentation thresholds based on the intensity and the magnitude of the intensity gradient, as described in^13^. The particle size distribution (PSD) was calculated from the segmented data using the continuous particle size distribution method^25^. The interface analysis between the different phases is performed as in^26^. The segmentation, interface analysis and PSD are computed using in-house Matlab and Python scripts. All plots were produced in MatLab 2020b while the 3D renderings in Fig. 1c and Figure S3 were done in ParaView 5.10 (Kitware, Inc).

### Tortuosity and effective conductivity simulations

To perform numerical simulations on the catalyst layer microstructure, a voxel-based mesh was generated by direct conversion of the microstructure voxels to mesh elements^27^. Thus, every mesh element had the same dimensions as the voxel resolution for the microstructure. The meshes were generated using the open-source library pyfcst which is a sub-library of the open-source package OpenFCST^28^.

The numerical simulations to compute the tortuosity and two-phase effective conductivity were performed using the open-source package OpenFCST. To compute the tortuosity for the different phases, a Laplace equation of the form:

1 $$\nabla \cdot \left({A}\nabla {x}\right) = 0$$

was used, where $A$ is the transport coefficient and $x$ is the scalar variable. $A$ and $x$ would correspond to the electronic conductivity and electronic potential for the catalyst and support phase, protonic conductivity and protonic potential for the ionomer phase and gas diffusivity and gas concentration for the void phase. The following boundary conditions were used:

2 $$\begin{aligned} & x = x^{in} \,{\text{on}}\,\Gamma_{1} \\ & x = x^{out} \,{\text{on}}\,\Gamma_{2} \\ & - A\nabla x \cdot \vec{n} = 0\,{\text{everywhere}}\,{\text{else}} \\ \end{aligned}$$

where ${x}^{in}$ and ${x}^{out}$ are the values of the independent variable $x$ at the inlet (${\Gamma }_{1}$) and outlet (${\Gamma }_{2}$) planes. Equation (1) was solved with the boundary conditions described in Eq. (2) to compute the total flux (${N}$) at the outlet. The effective property (${A}^{eff}$) was then computed from the total flux using:

3 $${\text{A}}^{{{\text{eff}}}} = {\text{N}}\frac{{\text{l}}}{{{\mathrm{a}}_{{{\text{cs}}}} \left( {{\text{x}}^{{{\text{in}}}} - {\text{x}}^{{{\text{out}}}} } \right)}}$$

where $l$ is the shortest distance separating the inlet and outlet planes and ${a}_{cs}$ is cross-section area of the outlet plane.

The tortuosity ($\uptau$) for the individual phases was then calculated as:

4 $$\uptau = \upvarepsilon \frac{{\text{A}}}{{{\mathrm{A}}^{{{\text{eff}}}} }}$$

where $\upvarepsilon$ is the phase volume fraction.

To compute the two-phase (both the catalyst and support layer together) effective electronic conductivity, modified meshes consisting of both phases with different material ids were generated. For the numerical simulations, the coefficient $A$, which corresponds to electronic conductivity ($\upsigma$), was assigned different values depending on the mesh material id.

## Results and discussions

### Image quality, resolution, and electron density determination

For the analysis of the electron density and phase fractions, a sub-volume of ~ 9 × 9x7 µm^3^ is extracted from the entire tomogram. Figure 1a shows a two-dimensional slice obtained from the extracted dataset where all phases are resolved.

Using the three-dimensional Fourier Shell Correlation curve with a half-bit threshold (FSC)^15,29^, we measure a half-pitch resolution of ~ 16.7 nm, close to the employed voxel size. Details of the FSC calculations can be found in section S2 of the supplementary materials. Therefore, any feature with a characteristic size smaller than 16.7 nm is not resolved and was neglected in the analysis.

After tomographic reconstruction, PXCT provides the three-dimensional distribution of the complex-valued refractive index^30^. Away from the absorption edges of the constituent materials, the 3D electron density distribution ${n}_{e}({\varvec{r}})$ can be obtained from the real part of the refractive index, $\delta \left({\varvec{r}}\right)$, by

5 $${{n}}_{{e}}\left(\varvec{r}\right)=\frac{2{\pi \delta }(\varvec{r})}{{\uplambda }^{2}{{r}}_{0}}$$

where $\lambda$ is the wavelength of the incident X-ray beam and ${r}_{0}$ is the classical radius of the electron. The electron density is then linked to the mass density as follows:

6 $$\uprho =\frac{{{n}}_{{e}}{M}}{{{N}}_{{a}}{Z}}$$

where M is the molar mass, ${N}_{a}$ is the Avogadro’s number and Z is the number of electrons in one molecule of the material. Therefore, if the chemical composition of the sample is known, ptychography can be used to map the mass density distribution in the sample and measure the variations compared to the expected values^31^.

Figure 1b shows the electron density histogram measured over the entire analyzed volume. Table 1 shows the values of electron density measured corresponding to the peaks related to each phase. The error in the table is computed by taking the electron density measured at the half-height of the maximum peak values of the histogram.

**Table 1:** Comparison between the measured mass density and theoretical density of the constituent materials.

Phase	Measured mass density [g/cm^3^]	Expected mass density [g/cm^3^]
Ionomer	2.1 ± 0.3	2.10^32^
TiO_2_	4.4 ± 0.7	4.23^18^
IrO_2_	8.0 ± 0.4	11.66^18^

As expected, the values related to the porosity are centered around zero, the negative electron density values are due to statistical fluctuation related to the image noise. The expected mass density of the titanium(IV) oxide phase, in the rutile form, lies within the interval of values obtained experimentally from ptychography, testifying that the TiO_2_ particles are mostly dense, presenting a negligible internal porosity.

In dry conditions, the commonly reported value for the density of dry Nafion membranes is around 2.1 g/cm^3^^32^. Considering that the ionomer employed has an equivalent weight $EW=1100 g/mo{l}_{S{O}_{3}}$ and a backbone length of $m= 6$^32^, the value calculated from the tomogram is very close to that of a bulk Nafion membrane. While it was hypothesized that the ionomer mass density can depend on the film thickness^33^, results suggest that the ionomer in the CL, for films with a thickness greater than ~ 17 nm, presents the same mass density as in the bulk membrane.

However, a considerably lower mass density is measured for the IrO_2_ phase than the theoretical value. This suggests the presence of nano-porosity within the IrO_2_ particles that are not resolved using PXCT. These nano-pores lead to the apparent reduced mass density and also contribute to additional surface roughness which cannot be resolved with the present resolution of 16.7 nm. Considering the ratio between the IrO_2_ theoretical and measured mass density and assuming that the nanopores are empty, we can estimate a 25% porosity for the IrO_2_ phase. However, this estimate might vary considerably in case the internal IrO_2_ porosity is partially or fully filled with the ionomer phase. Further analyses using more sensitive techniques such as mercury intrusion porosimetry (MIP) or TEM tomography will be required to clarify this point.

### Phase fractions, ink composition and particle size distributions

**Figure 2:**
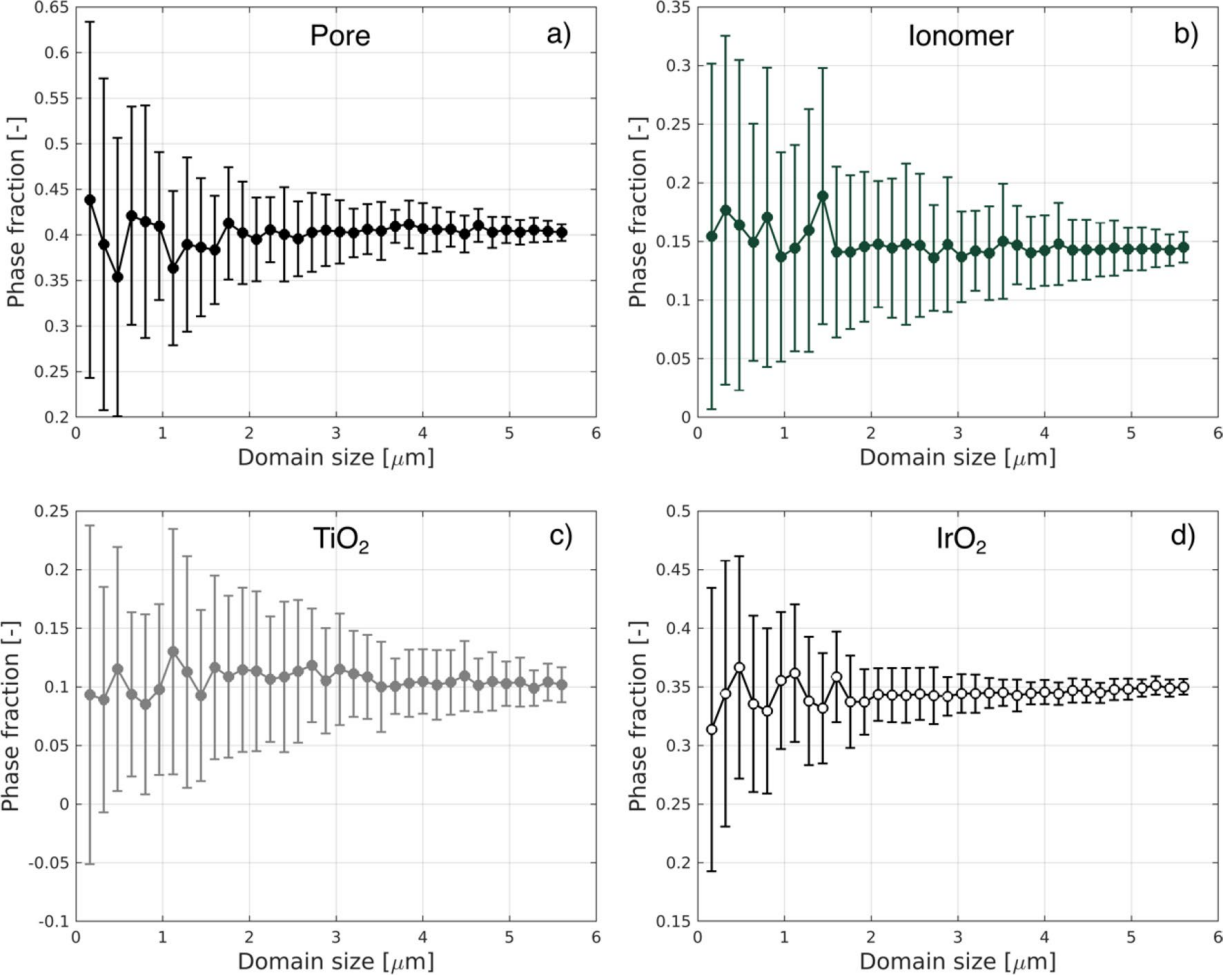
Representative volume analysis performed calculating the average phase fraction and standard deviation of 50 sub-volumes extracted from the entire dataset from random locations. (**a**) Pore, (**b**) Ionomer, (**c**) TiO_2,_ and (**d**) IrO_2_.

The volume fractions of IrO_2_, TiO_2_, ionomer, and pores are calculated from the segmented data, using the biggest rectangular volume (~ 9 ×  9 × 7 µm^3^) extractable from the quasi-cylindrical pillar. A representative elementary volume (REV) analysis has been performed to ensure reliability of reported values. For the analysis, 50 sub-volumes of varying sizes have been sampled from the entire dataset from random locations. For each sub-volume set, the average value of the phase fraction and its standard deviation have been computed. The results of the representativeness analysis are presented in Fig. 2.

Figure 2 shows that, for volumes exceeding 5 × 5 × 5 µm^3^, the relative standard deviation ranges from a minimum of less than 1% (Pores) to a maximum of 7% (TiO_2_) suggesting that the volume investigated can be considered representative for further analysis.

The phase fractions in the electrode can be compared to the theoretical volume fractions computed from the ink composition (used for the spray coating procedure), the density of the constituent materials, and the thickness of the catalyst layer. The volume fractions for the IrO_2_ and TiO_2_ can be deduced from the catalyst loading employed. The average thickness of the catalyst layer was determined by averaging the distance between the Nafion membrane and the carbon coating (electrode surface), as explained in section S4 of the supplementary materials. Using this procedure, we could calculate an average thickness of 9.5 µm, obtained by averaging the catalyst layer thickness measured at several locations. From the catalyst loading (${L}_{cat}$), the thickness of the electrode (${t}_{cat}$), and the average catalyst density (${\rho }_{cat}$), the volume fraction (${V}_{cat}$) can be calculated as:

7 $${{V}}_{{cat}}= \frac{{{L}}_{{cat}}}{{\rho }_{{cat }}\ {{t}}_{{cat}}}$$

The expected average catalyst density (${\rho }_{cat}$) is 9.5 g/cm^3^, determined assuming that the catalyst consists of iridium(IV) oxide and titanium(IV) oxide as follows: Knowing the weight percentages of the components (86.9 wt% IrO_2_ and 13.1 wt% TiO_2_) and the density of the materials (11.7 g/cm^3^ for IrO_2_ and 4.23 g/cm^3^ for TiO_2_), we can calculate the IrO_2_ and TiO_2_ volume fraction of 27% and 11% respectively. Similarly, the same equation can be applied to calculate the ionomer volume fraction:

8 $${{V}}_{{ion}}= \frac{{{L}}_{{ion}}}{{\rho }_{{ion }}\ {{t}}_{{cat}}}$$

As discussed in the previous section, using our measured density of ${\rho }_{ion}=2.1\,{\mathrm{g}}/{\mathrm{cm}}^{3}$, we can finally calculate an ionomer volume fraction of 22% with a remaining porosity of 42%.

Table 2 shows that the calculated TiO_2_ and pore phase fraction corresponds quite well with the expected values based on the ink composition. However, from the tomographic data, the IrO_2_ volume fraction is overestimated while the measured ionomer phase fraction is considerably lower than the one expected.

**Table 2:** Comparison between the measured phase fraction and the expected values based on the ink composition. All reported phase fractions have been rounded to the closest integer.

Phase	Phase fraction from ink [%]	Measured phase fraction [%]
Ionomer	22	15
TiO_2_	11	11
IrO_2_	27	36
Pore	42	39

The overestimation of the IrO_2_ volume fraction is likely due to the hypothesized internal nano-porosity, which is neglected in the segmentation, resulting in a higher IrO_2_ volume fraction value. Similarly, the discrepancy observed for the ionomer phase fraction could be also explained assuming that not all ionomer phase is detected. The ionomer could be present as a thin film (< 10 nm thick) covering the surface of the catalyst particles, in this form it would not be detected by the segmentation. Furthermore, taking into account the nano-porosity hypothesized for the IrO_2_ phase, the ionomer could be present in between the nanoparticles.

**Figure 3:**
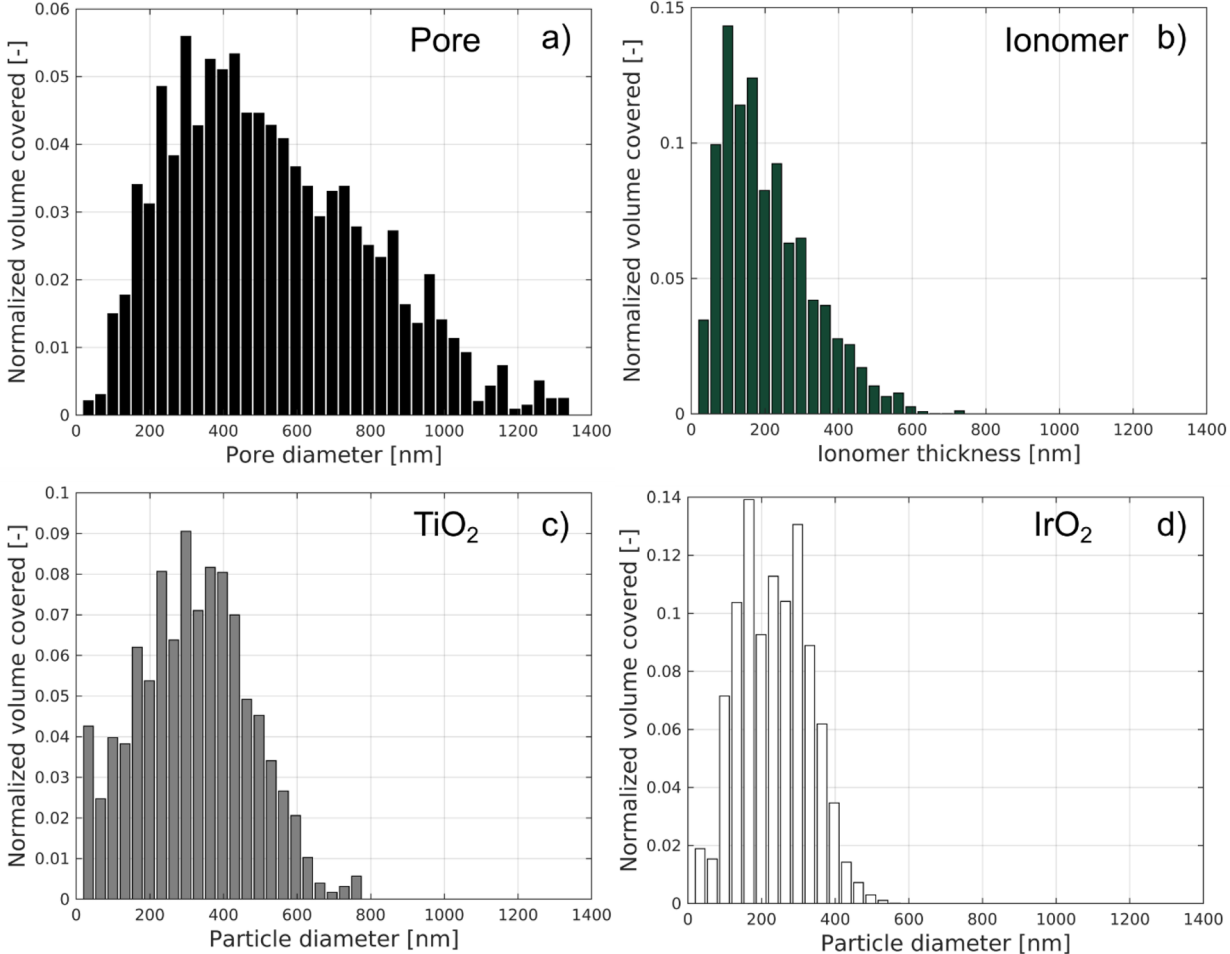
(**a**) Pore size distribution; Particle size distribution (local thickness) for the (**b**) ionomer, (**c**) TiO_2_ and, (**d**) IrO_2_ phase.

Figure 3 shows the continuous pore and particle size distribution (cPSD) of the electrode constituent phases. The pore phase (Fig. 3a) has a broad distribution, with a maximum detected pore diameter of ~ 1.2 µm. Figure 3b shows the particle size distribution of the ionomer phase, corresponding to the local thickness of the ionomer across the analyzed volume. The majority of the detectable ionomer phase presents a thickness between 100 and 300 nm. However, large agglomerates with a diameter > 400 nm are also present. These agglomerates are found in between catalyst domains, connecting the different IrO_2_/TiO_2_ particles. Support particles (TiO_2_) present a wide range of diameters (0–800 nm), with a peak around 300 nm (Fig. 3c). As shown in Fig. 1, the IrO_2_ forms both shells around the TiO_2_ and small self-standing particles. Figure 3d shows that the majority of the particles present a diameter ranging from 100 to 300 nm, i.e. smaller sizes as compared to the TiO_2_ phase.

### Interface areas

The microstructure of the catalyst layer determines the accessibility of the catalyst and is crucial for electrochemical reactions. The active area is usually referred to as the electrochemically active surface area (ECSA) and it is an important parameter for determining the overall activity of the catalyst layer, hence influencing the PEWE cell performance. In the tomographic data, the support phase (TiO_2_) shares its surface only with the IrO_2_ phase and therefore is not reported in the calculations. From the tomograms, the values for the different interface and surface areas are shown in Table 3. All values are reported as volume-specific surface areas and are normalized by the volume of the analyzed region.

**Table 3:** Summary of the measured surface area, interface areas, and total TPBs density.

Phases surface and interfaces	Normalized Area [µm^2^/µm^3^]
Pore total surface area	2.9
Ionomer total surface area	3.0
IrO_2_ total surface area	3.6
Pore/Ionomer interface area	1.2
Pore/IrO_2_ interface area	1.7
IrO_2_/Ionomer interface area	1.9

**Figure 4:**
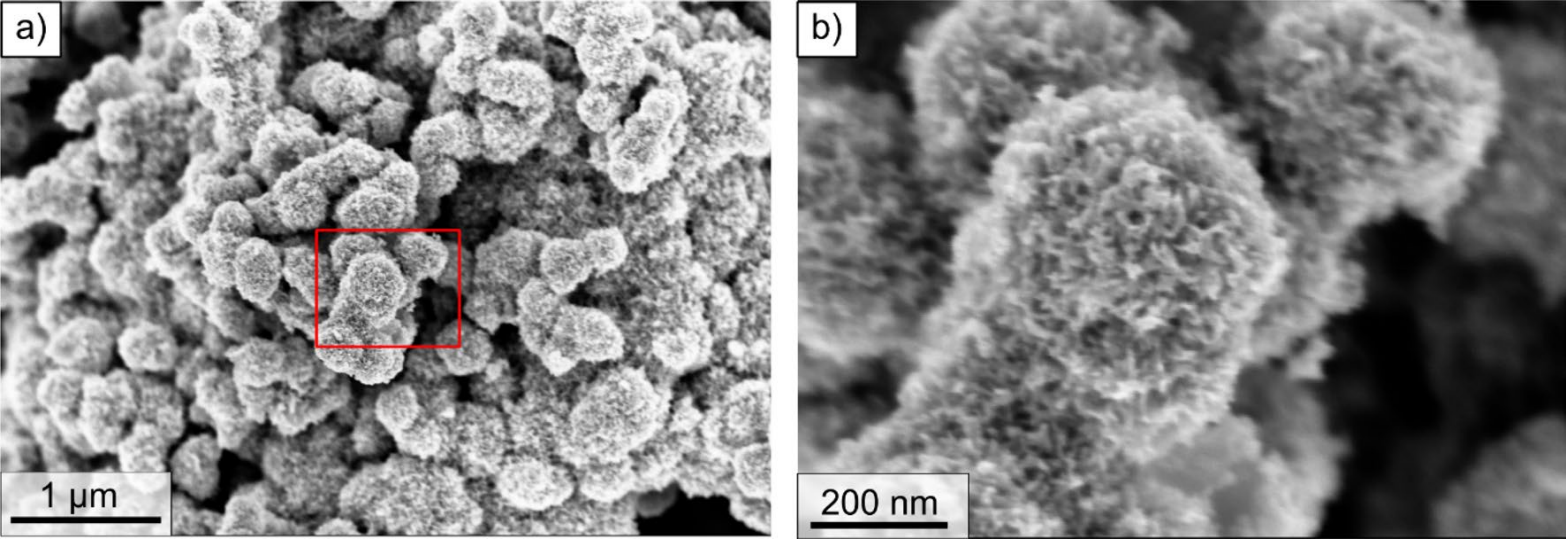
(**a**) SEM micrographs of the pristine IrO_2_/TiO_2_ catalyst powder employed in this work; (**b**) magnified view of the region highlighted by the red rectangle in (**a**).

Assuming that all IrO_2_ surface is active, to calculate the ECSA, the following formula has been used:

9 $${ECSA}={a}*{{t}}_{{cat}}*{{L}}_{{cat}}^{-1}$$

considering a total IrO_2_ volume-specific surface area of $a=3.6 {10}^{-6}$ m^-1^, an electrode thickness of ${t}_{cat}=9. 5\, \upmu{\text{m}}$ and a nominal catalyst loading of ${L}_{cat}=3\,{\mathrm{mg}}\,{\mathrm{cm}}^{-2}$, we calculate an ECSA of 1.2 m^2^/g. Interestingly, this value is close to what was reported in^6^, where the authors calculated a similar ECSA for an Ir-Ru catalyst using FIB-SEM tomography. However, previous studies^18,34^ have reported values in the order of 29–31 m^2^/g. This discrepancy is likely due to the limited spatial resolution, which makes the technique not able to fully resolve the roughness of the IrO_2_ on the surface of the TiO_2_. The roughness of the IrO_2_ surface, below the current resolution, can be seen qualitatively in Fig. 4, but cannot be quantified.

Furthermore, Table 3 shows that the ionomer that can be resolved in the tomographic data, does not fully cover the catalyst particles. The IrO_2_/Ionomer interface is measured to be 1.9 µm^−1^, representing ~ 53% of the total IrO_2_ surface area.

Figure S3 in the supplementary materials visually shows the different interfaces, highlighting in red the IrO_2_/ionomer interface with ionomer coverage > 17 nm. Considering the limited resolution, the possibility of a thin ionomer layer (< ~ 17 nm) at the surface of the catalyst layer seems probable, as this would be in line with the missing ionomer volume fraction observed (see Table 2). Based on the reconstructed CL volume, it can be observed that the ionomer forms films (~ 32–64 nm thick) covering the IrO_2_ surface as well as large agglomerates forming bridges between different particles. Such blob structures of ionomer in the CL are characteristic of the spray- or print-coating method and have also been observed for polymer electrolyte fuel cell CLs^35^. Although ionomer thin films (< ~ 17 nm) might be covering the IrO_2_ surface, the uneven ionomer distribution achieved using spray coating might also result in only partial coverage of the IrO_2_ surface by the ionomer. Higher-resolution imaging techniques such as TEM tomography, could provide further insights into the thin film ionomer distribution on the IrO_2_ surface and provide a more accurate estimate of the IrO_2_/Ionomer interface area.

### Connectivity and transport properties

In the catalyst layer, reactants and products have to be transported to and from the active sites. The morphology of the microstructure is crucial for the transport of each species. Table 4 shows the tortuosity and effective conductivity/diffusivity values for all phases.

**Table 4:** Summary of the measured connectivity, tortuosity and, effective transport property for each phase.

Phase	Connectivity [–]	Tortuosity [–]		${A}^{eff}/A$[–]	
		In-plane	Through-plane	In-plane	Through-plane
TiO_2_	0	–	–	–	–
IrO_2_	0.99	3.5	4.2	0.09	0.08
Pore	0.99	2.3	2.8	0.17	0.15
Ionomer	0.95	6.1	7.2	0.02	0.01

The connectivity of the support phase (TiO_2_) is 0, testifying that the TiO_2_ particles are fully isolated. The catalyst and pore phase present almost complete connectivity. Interestingly, the ionomer phase also has a high connectivity (95%), despite the smaller phase fraction when compared to the IrO_2_ and pore phase.

The tortuosity values for each phase are calculated using Eq. (4). For all three connected phases, the tortuosity values vary slightly between the in-plane and through-plane directions, revealing a slight anisotropy in the electrode. Interestingly, results indicate that, for all cases, in-plane transport is slightly more efficient than the through-plane. This effect is likely the result of the morphological arrangement obtained via spray-coating and might be different for other deposition techniques. Further studies are required to elucidate the effect of the deposition technique on CL morphology.

The effective conductivities are reported as the ratio between the calculated values and the bulk transport property of the respective phase. For the ionomer protonic conductivity, several values have been reported in the literature as a function of water content, temperature, and film thickness^32^. However, for conditions relevant for electrolysis applications it can be estimated to be between 0.1 and 0.2 S/cm^18^. The reported value for the apparent conductivity of the IrO_2_ is ~ 64 S/cm^5^.

Therefore, using the ratios shown in Table 4, we can estimate that for the morphology examined in this work, the effective protonic conductivity of the catalyst layer to be in the order of ~ 10^–3^ S/cm while the electrical conductivity is in the order of ~ 5 S/cm. The value for the electrical conductivity is similar to what was reported by Schuler et al.^36^, based on a similar catalyst layer, experimentally measured with a four-point probe setup in dry conditions.

Therefore, based on the microstructure analysis, we conclude that for the analyzed structure under dry conditions, the electrical conductivity of the catalyst layer is several orders of magnitude higher than the ionic conductivity (when assuming the conductivity of the hydrated ionomer). This observation is in agreement with Bernt et al.^18^ and Schuler et al.^36^.

However, in presence of liquid water, the expansion and deformation of the ionomer might lead to the disruption of the IrO_2_ percolating network, leading to a decrease in overall electronic conductivity. This effect was already hypothesized in^36^ and will be examined in a future in-situ tomography work. Furthermore, imperfections in the segmentation due to limits in resolution might lead to errors in the reported conductivity values. For example, the hypothesized porosity in the IrO_2_ is neglected, leading to an overestimation of the CL electronic conductivity. However, the good agreement with literature data^36^ hints that this effect has a small impact on the simulation results.

### Catalyst layer conductivity as a function of support conductivity

In PEWE, to reduce the precious metal loading, high surface area supports are normally used. Support materials promote the dispersion of the IrO_2_ nanoparticles and reduce the agglomeration of the active catalyst. However, due to the acidic environment and the high anodic potential, only some oxides or carbides offer the required stability for long-term operation. With the aim of finding the most conducting support while preserving the required chemical stability, various materials have been researched such as tantalum-doped TiO_2_^37^, niobium-doped TiO_2_^38^, or antimony-doped SnO_2_ (ATO)^39^.

However, the influence of the support conductivity on a practical CL is still unclear and depends on parameters like Ir loading, IrO_2_ connectivity, and overall CL morphology. For example, Polonsky et al.^40^ have reported that the support conductivity is not critical and non-conductive materials might also be considered as a support for IrO_2_ catalyst.

**Figure 5:**
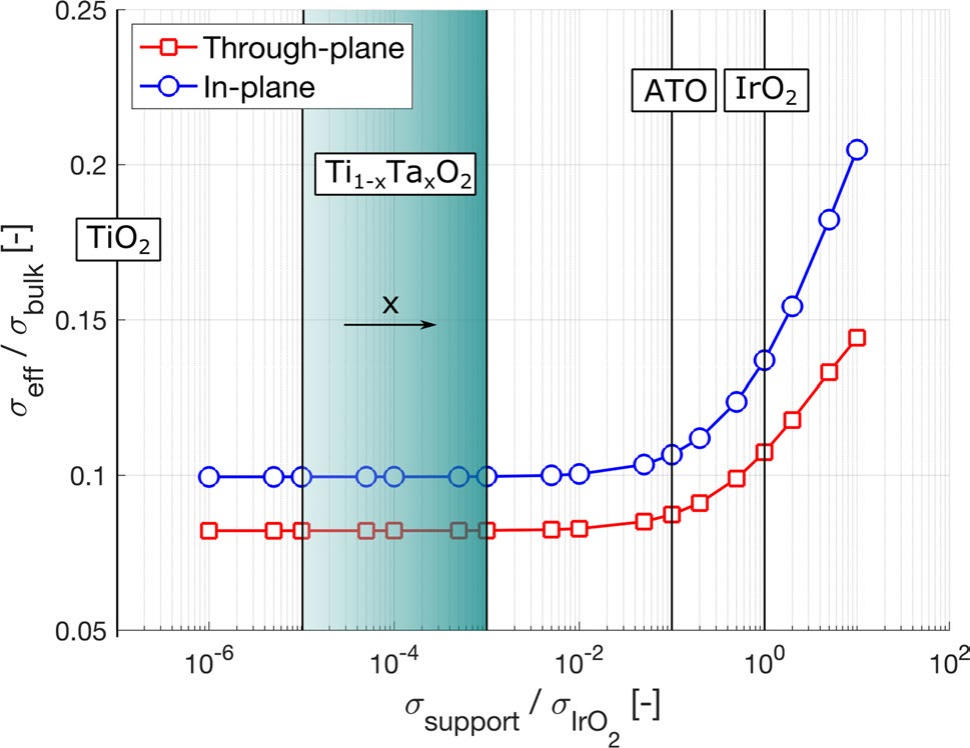
Electrical conductivity of the catalyst layer as a function of support conductivity, for both the in-plane and through-plane direction. The y-axis shows the computed electrical conductivity, normalized by the IrO_2_ bulk value. On the x-axis, the conductivity of the support material (normalized by the IrO_2_ conductivity) is varied, spanning eight orders of magnitudes. The normalized conductivity of the pure TiO_2_ is 10^–7^.

Having access to three-dimensional data and spatial information on the IrO_2_/TiO_2_ morphology, we can calculate the catalyst layer's overall electrical conductivity. Figure 5 shows the results of the simulations for both the through-plane and in-plane directions. In Fig. 5, the x-axis represents the ratio between the support conductivity (varied in the simulation) and IrO_2_ intrinsic apparent conductivity^5^. The y-axis shows the ratio between the computed effective CL conductivity and the IrO_2_ bulk conductivity.

In this study, the support conductivity is varied across eight orders of magnitudes, to simulate the effect of employing some of the previously reported support materials on the overall CL conductivity. As examples, the values of ${\sigma }_{support}/{\sigma }_{IrO2}$ for the pure TiO_2_ and tantalum-doped TiO_2_^37^, ATO^41^ and IrO_2_ are indicated in Fig. 5. For simplicity, only the order of magnitude is considered. For the tantalum-doped TiO_2_, the range of conductivity values indicated by the green rectangle corresponds to the different levels of Ta doping, with x going from 0.05 to 0.3.

From Fig. 5, the following observations can be drawn:

1. Overall, changing the support connectivity of eight orders of magnitudes results only in doubling the electric CL connectivity.
2. The curve is relatively flat for ratios below 10^–1^ and presents a steep increase for values of support connectivity exceeding the one of IrO_2_
3. A pure IrO_2_ catalyst would have only a ~ 30% higher electrical conductivity than the case with the support.
4. Using the majority of the support materials reported in literature, the conductivity of CL will only be marginally affected.

Generalizing from the catalyst materials and structure morphology employed in this work, for any CL where the percolation of the IrO_2_ is ensured, any high-surface area support material can be employed, even if fully electrically insulating. Furthermore, the use of the support is always advised since the benefits in reducing Ir loading and potentially increasing the CL active area greatly outweigh the marginally reduced conductivity. Finally, even at high loading and with a full percolating IrO_2_, the CL conductivity can be improved only using materials with a higher conductivity compared to the IrO_2_.

The importance of the support conductivity will be more pronounced for CLs with ultra-low loadings where a fully connected IrO_2_ percolating network might not exist.

## Conclusions

In order to characterize the catalyst layer morphology, we employed the first three-dimensional reconstruction of the OER catalyst TiO_2_-supported IrO_2_ using high-resolution ptychographic tomography. With the unique capabilities of the technique, including the high spatial resolution, low noise, and high sensitivity to small variations in electron density, all phases (TiO_2_, IrO_2_, ionomer and pore) were clearly resolved.

From the analysis of the electron density, we estimated that the TiO_2_ particles are dense and they are present in the rutile form. The mass density of the ionomer (in the catalyst layer structure) is confirmed to be close to the bulk Nafion properties. The IrO_2_ phase presents an electron density lower than the expected value, indicating that the IrO_2_ likely has a nanoporosity below the limit of the spatial resolution of ptychographic tomography, as shown by the SEM analysis.

The analyzed volume of 9 × 9x7 µm^3^ is proved to be representative for all phases. The phase fraction calculations present a discrepancy to the expected values derived from the catalyst layer ink composition. The overestimation of segmented IrO_2_ volume fraction is caused by the presence of nanoporous IrO_2_ as confirmed by SEM images. The analysis of the continuous pore and particle size distribution reveals that the void phase presents a broader size distribution compared to the other phases.

For an ionomer to catalyst content of 11 wt%, the majority of the ionomer phase presents a thickness between 100 and 300 nm, however, agglomerates larger than 400 nm are also present, showing that the ionomer does not form a uniform layer covering the surface of the IrO_2_ but also interconnects catalyst agglomerates.

The analysis of the phases’ interface areas reveals a total catalyst surface of 1.2 m^2^/g, a factor of ~ 25 lower than reported in literature, testifying that the IrO_2_ nanoscale surface roughness presents a rugosity below the available spatial resolution of 16.7 nm. Furthermore, in the segmented volume, only ~ 53% of the IrO_2_ surface appears covered by the ionomer with a layer thickness of > 16 nm, hinting at the possibility that the rest of the catalyst surface is not fully covered by the ionic conductive phase or covered by a thinner layer.

Results show that the pore phase has the least tortuosity while the ionomer with 7.1 presents the highest values. The effective conductivities computed from the tomographic data are comparable with previous experimentally reported values and indicate that, in the structure with a non-swollen ionomer, the ionic conductivity is the limiting component, even if the ionomer is in a well-conducting state.

For catalyst layers having similar morphology, IrO_2_ loadings, and IrO_2_/support ratios, the analysis of the support conductivity shows that the presence of the support phase only marginally affects the overall CL conductivity. A pure IrO_2_ catalyst with the same morphology possesses only a ~ 30% higher electrical conductivity. Furthermore, varying the support conductivity below the IrO_2_ value has only a negligible impact on the overall CL conductivity, proving that even electrically-insulating support materials can be used. Finally, for a catalyst structure with low loading and a non-percolating IrO_2_ phase, a support material with electrical conductivity in the range of IrO_2_ is required to obtain similar CL conductivities.

This study provided not only insights into state-of-the-art catalyst layers via synchrotron-based PXCT and guidance of rational catalyst layer design but also provides a novel tool for analysis of future generation low-loaded CL microstructures.

## Supplementary Information

Supplementary Information.

## Data Availability

The research data produced in this article will be made available upon request. Please contact Salvatore De Angelis (sdea@dtu.dk) or Felix Büchi (felix.buechi@psi.ch) for getting the data or for further information.
