# Supplementary material for: Understanding the microstructure of a core–shell anode catalyst layer for polymer electrolyte water electrolysis
Source: Sci Rep. 2023 Mar 15;13:4280. doi: 10.1038/s41598-023-30960-x (PMC10017760; doi:10.1038/s41598-023-30960-x)
Supplement: Supplementary file 1 — Supplementary Information. [file 41598_2023_30960_MOESM1_ESM.docx]

**Supplementary Materials**

***Understanding the microstructure of a core-shell anode catalyst layer for polymer electrolyte water electrolysis***

Salvatore De Angelis^1^, Tobias Schuler^1^, Mayank Sabharwal^1^, Mirko Holler^2^, Guizar Sicairos Manuel ^2^, Elisabeth Müller^3^, Felix N. Büchi^1^

^1^ Electrochemistry Laboratory, Paul Scherrer Institute, CH-5232 Villigen PSI, Switzerland.

^2^ Swiss Light Source, Paul Scherrer Institute, CH-5232 Villigen PSI, Switzerland

^3^ Electron Microscopy Facility at PSI, CH-5232 Villigen PSI, Switzerland

**S1. Lift-out procedure at the FIB-SEM**

In this work, samples were produced using the lift-out technique, extracting the sample from a state-of-the-art self-sprayed anodic catalyst layer (CL). The catalyst-coated membrane was secured on a standard SEM holder, with the anodic catalyst layer facing the electron and ion beam. The lift-out procedure can be summarized in six steps:

1. A protective layer of carbon is formed by Ga-induced carbon deposition on top of the area from which the specimen shall be prepared for the ptychographic tomography measurement. This avoids excessive Ga-implantation into the specimen during further preparation. For C-deposition a beam of 200pA at 30kV was used with a dwell time of 0.2μs.
2. The focused ion beam is then used to mill away the material around the area of interest, isolating a rectangular sample (16x16 µm^2^). This was done with a Ga-beam accelerated with 30keV and a beam intensity of 3nA.
3. The rectangular area is undercut to detach it from the bulk Nafion membrane, keeping it still attached to bulk material by a small bridge (Figure S1 a).
4. A tungsten needle is introduced by a micro-manipulator and then attached to the sample using Ga-induce carbon-deposition via a gas injection system (Figure S1 b)
5. With the needle attached, the sample is cut out from the bulk material using the focused-ion beam, removing the small bridge . The sample is then lifted for positioning on the OMNY sample holder ^1^ (Figure S1 c)
6. The sample is transferred onto the OMNY pin, attached via carbon deposition and the needle is then cut free with the focused ion beam. The original rectangular shape is refined using the focused ion beam (with a 30keV beam of 700pA intensity), milling the sample edges and producing the final near-octagonal cross-section visible in Figure S1 d.


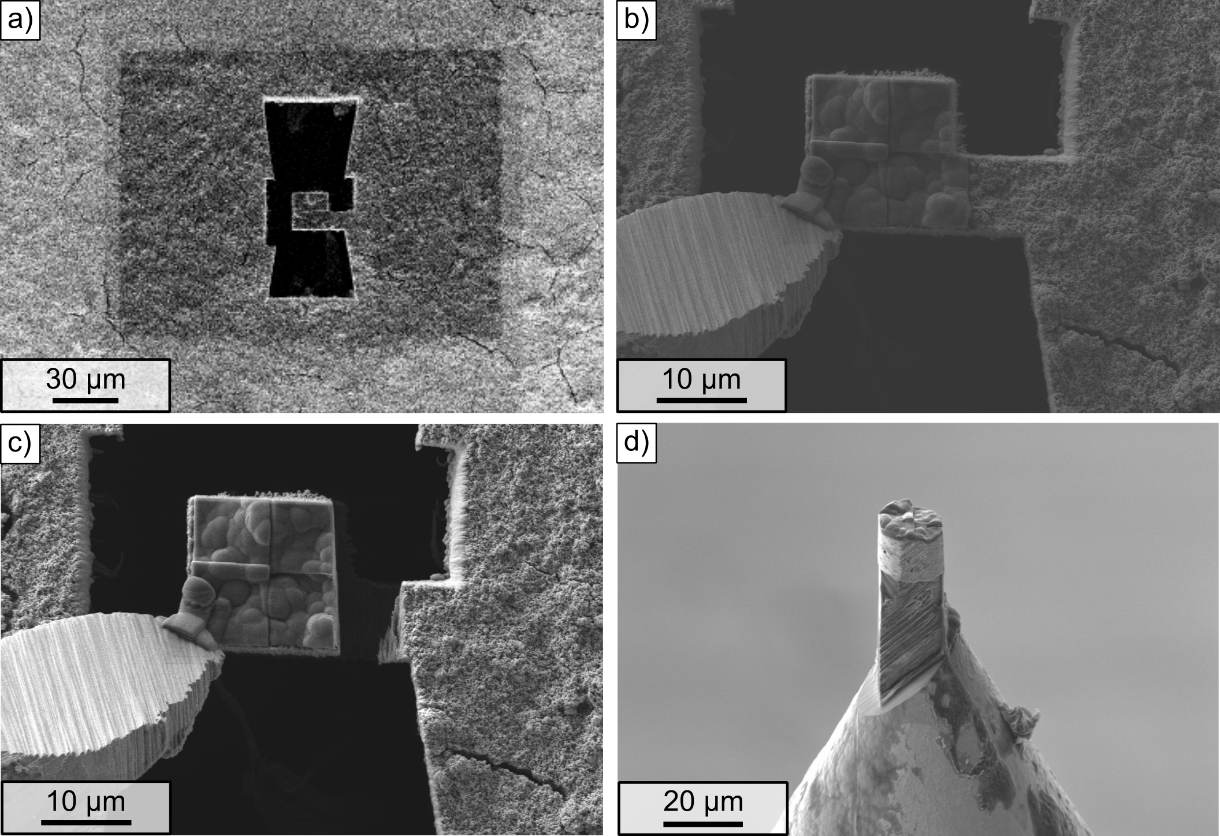


**Figure S1. Summary of the lift-out procedure. a) SEM micrograph of the milling geometry visualized from the top; b) Tungsten needle attached to the sample; c) sample detached from the bulk material; d) Final sample mounted on the OMNY sample holder.**

The entire procedure was conducted in a Zeiss NVision 40 FIB/SEM instrument.

**S2. Fourier shell correlation method**

To estimate the tomogram resolution, the dataset including 1550 projections, was divided in half to generate two independent tomograms from half the number of original projections. From the two independent tomograms, a correlation in the Fourier domain is obtained and the resolution is estimated by the intersection with the ½ bit threshold curve, as described in ^2^.

Figure S2 shows the FSC curve and its intersection with the threshold curve. The estimated resolution is 16.71 nm, with a voxel size of 16.55 nm.


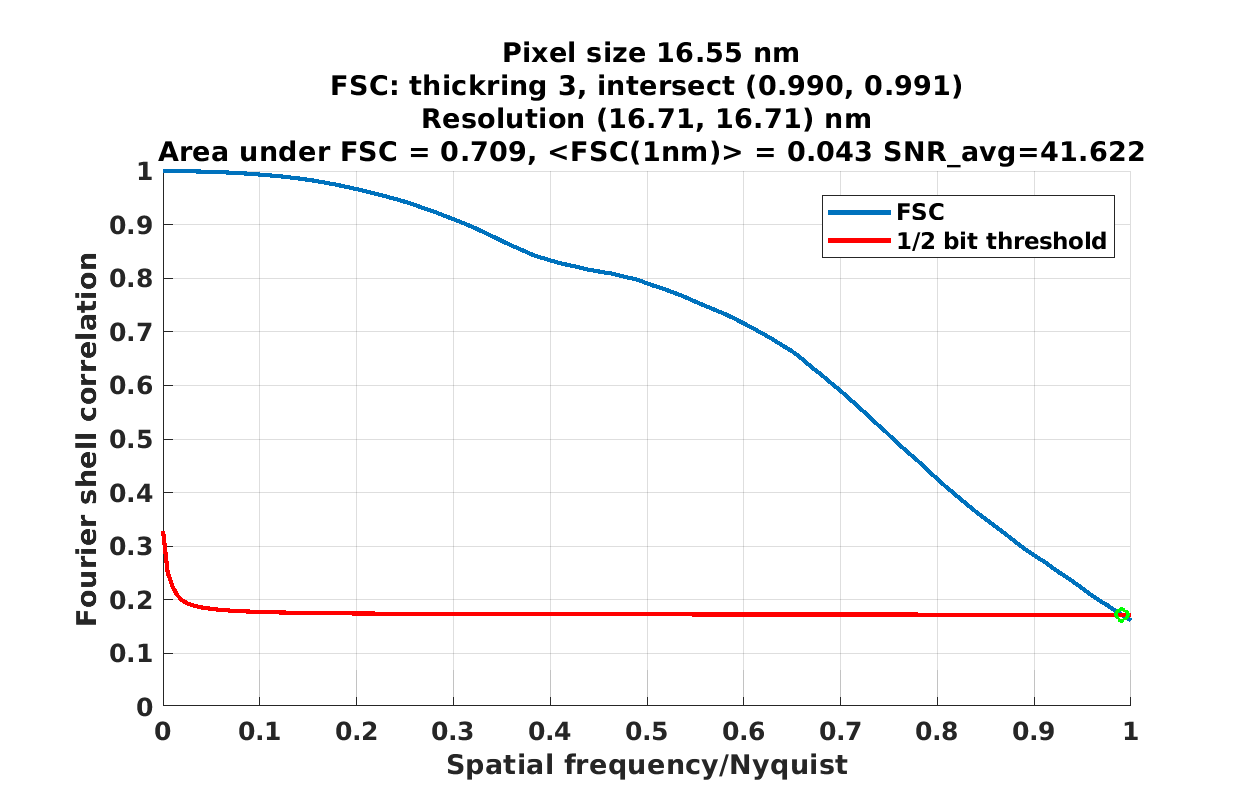


**Figure S2. Fourier shell correlation plot. The resolution is estimated using the ½ bit threshold criteria.**

**S3. Visual representation of the IrO_2_/Ionomer interface and triple-phase boundaries**

Figure S3 visually shows the different interfaces, highlighting in red the detected IrO_2_/ionomer interface while presenting the Pore/IrO_2_ in gray. Given the limits of the resolution (< 17 nm), if thin ionomer films are present on the IrO_2_ particles, the gray area in Figure S3 shows the fraction of IrO_2_ surface covered with either ionomer of thickness < 17 nm or being in direct contact with pores.

**
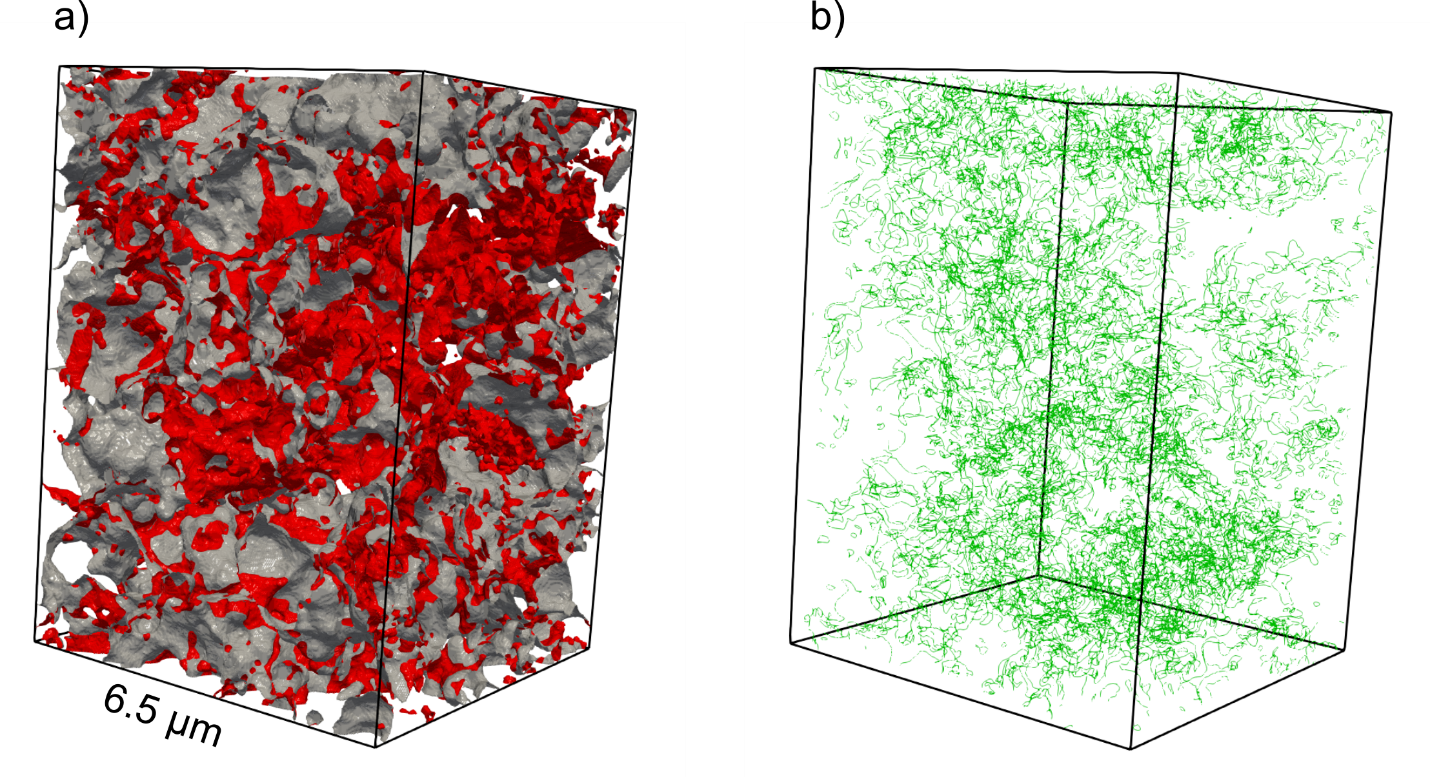
**

**Figure S3. Three-dimensional rendering of the IrO_2_ surface, from a sub-volume extracted from the entire dataset. The red areas highlight the Ionomer/IrO_2_ interface while the gray areas highlight the interface with either ionomer layers < 17 nm thick or with pores. The 3D rendering was made in Paraview 5.10 (Kitware, Inc., https://www.paraview.org).**

**S4. Anodic catalyst layer thickness estimation**

To estimate the average thickness of the electrode, we acquired a low-resolution scan where the entire thickness of the CL is in the field of view. Figure S4 shows an exemplary two-dimensional slice obtained from the entire tomogram, where the CL, the Nafion membrane (upon which the electrode is sprayed), and the protective carbon layer (deposited for the sample preparation explained in Section S1) are within the field of view. To calculate the average thickness, the CL thickness is measured in nine different locations (e.g red lines in Figure S4), for five different slices.


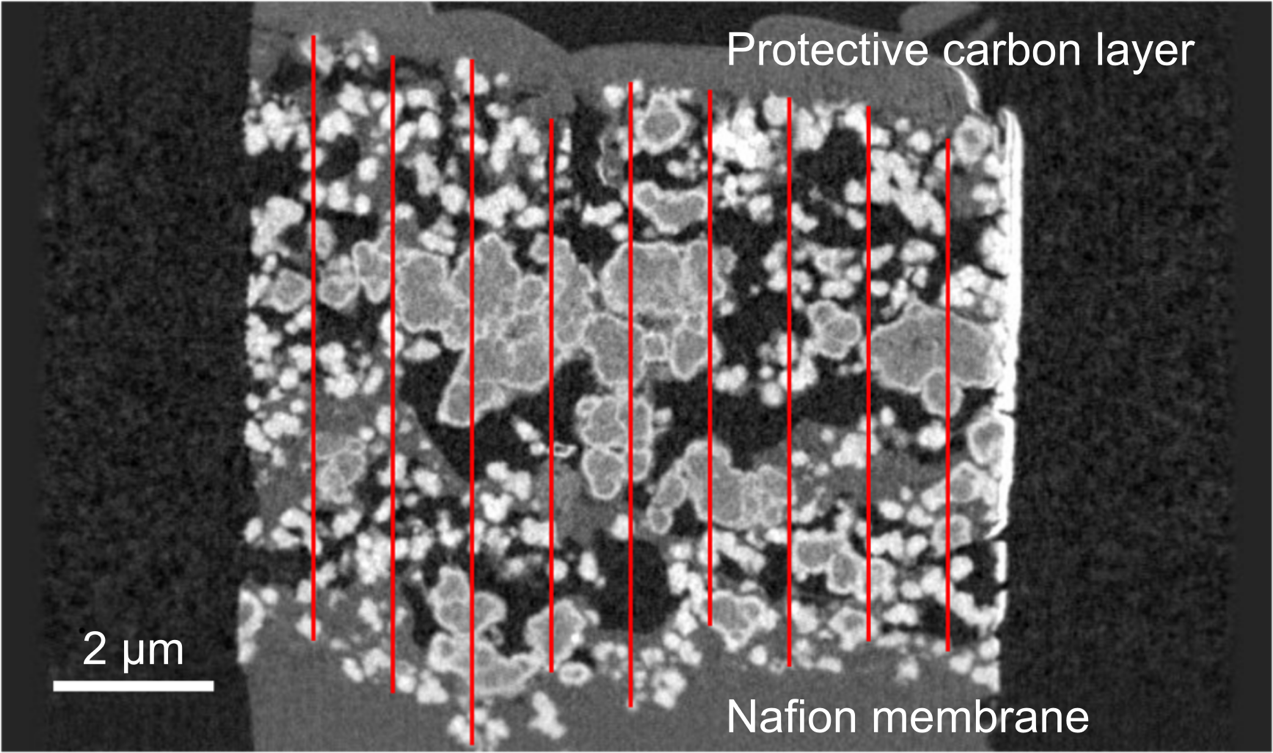


**Figure S4. Two-dimensional slice obtained from a low-resolution tomogram, where the entire thickness of the electrode is in the field of view. The red lines indicate the locations where the thickness was measured.**

**References**

1. Holler, M. *et al.* OMNY PIN—A versatile sample holder for tomographic measurements at room and cryogenic temperatures. *Review of Scientific Instruments* **88**, 113701 (2017).

2. Holler, M. *et al.* X-ray ptychographic computed tomography at 16 nm isotropic 3D resolution. *Scientific Reports* **4**, (2014).
